# Vimentin Intermediate Filaments as Potential Target for Cancer Treatment

**DOI:** 10.3390/cancers12010184

**Published:** 2020-01-11

**Authors:** Katerina Strouhalova, Magdalena Přechová, Aneta Gandalovičová, Jan Brábek, Martin Gregor, Daniel Rosel

**Affiliations:** 1 Department of Cell Biology, Charles University, Viničná 7, 12843 Prague, Czech Republic; strouhak@natur.cuni.cz (K.S.); gandaloa@natur.cuni.cz (A.G.); brabek@natur.cuni.cz (J.B.); 2 Biotechnology and Biomedicine Centre of the Academy of Sciences and Charles University (BIOCEV), Průmyslová 595, 25242 Vestec u Prahy, Czech Republic; 3 Laboratory of Integrative Biology, Institute of Molecular Genetics of the Czech Academy of Sciences, 14220 Prague, Czech Republic; magdalena.prechova@img.cas.cz

**Keywords:** vimentin, EMT, invasion, mechanotransduction, cell adhesion, cancer treatment, cancer drugs, amoeboid, mesenchymal

## Abstract

Intermediate filaments constitute the third component of the cellular skeleton. Unlike actin and microtubule cytoskeletons, the intermediate filaments are composed of a wide variety of structurally related proteins showing distinct expression patterns in tissues and cell types. Changes in the expression patterns of intermediate filaments are often associated with cancer progression; in particular with phenotypes leading to increased cellular migration and invasion. In this review we will describe the role of vimentin intermediate filaments in cancer cell migration, cell adhesion structures, and metastasis formation. The potential for targeting vimentin in cancer treatment and the development of drugs targeting vimentin will be reviewed.

## 1. Introduction

Intermediate filaments (IFs) [1], the most heterogeneous component of the metazoan cytoskeleton, are assembled from one or more highly conserved IF protein encoded by more than 70 genes in humans [2,3]. Based on the protein structure, sequence homology, and expression pattern, the IF protein superfamily is sub-classified into six different gene families. Apart from lamins (Type V; encoded by three genes), which are restricted to the nuclear compartment, all other families (Types I–IV and VI) are found in the cytoplasm. IFs Type I and II consist of two groups of keratins (encoded by 54 genes) that are predominantly expressed by epithelia. Individual cell types express unique IF signatures of more than five different IF proteins, including at least two cytoplasmic IFs and two to three nuclear lamins. In recent decades many IF proteins have been implicated in the regulation of 2D and 3D cell migration [4,5,6], however, to date, only vimentin (Type III) is widely accepted as a major migration enhancer [7].

The expression and assembly of IFs is tightly regulated in a tissue-, differentiation-, and context-dependent manner. It is appreciated that oncogenic transformation alters the cell type-specific IF signature, typically leading to upregulated expression of vimentin IFs [8]. Although it is hard to unravel cause–effect relations, there is accumulating evidence that IFs act as both target and effector, thus constituting a potential target for cancer treatment.

However, appealing the idea of targeting intermediate filaments as a cancer treatment may be, it remains a challenging task. Unlike the other cytoskeletal networks, microtubules and the actin cytoskeleton, intermediate filaments are not yet easily targetable by IF protein-specific drugs. As evidenced by recent discoveries discussed in the following text, intermediate filaments, particularly vimentin, have increasing implications in cancer progression, cancer cell migration, and invasion, and there is an intensified need for specific targeting of vimentin. 

In this review, we focus mainly on tumor marker vimentin IFs in the context of epithelial-to-mesenchymal transition (EMT), cell migration, and invasion. Lastly, we discuss the current possibilities of using vimentin as a potential drug target for cancer treatment.

## 2. Vimentin in EMT

Vimentin is expressed from early stages of embryonic development in highly plastic mesenchymal cells. During later development, it becomes excluded from keratin-expressing epithelia. Following the same paradigm, the oncogenic transformation of epithelial cells results in an upregulation of vimentin and subsequent loss of keratin [8]. As the first step of becoming migratory, cells undergo EMT, a switch from epithelial polarity to front–rear polarity and loosen cell–cell junctions. Indeed, vimentin is widely used as a canonical marker of EMT reprogramming, associated with the acquisition of a migratory and invasive tumor cell phenotype [4]. As such, vimentin is abundantly expressed in many tumor types (for review see [9]), where its expression correlates with their aggressiveness and poor clinical outcome. Over-expression of vimentin in epithelial cells has been shown to be sufficient for cells to adopt the elongated shape typical of mesenchymal cells. This is followed by the reorganization of the actin and microtubule cytoskeletons [10], the internalization of desmosomes [11], and the rearrangement of keratin IFs [12]. Conversely, downregulation of vimentin not only hampers the migration of a large variety of tumor cell lines [4] but also partially restores their epithelial phenotype [13].

EMT is governed by several signaling pathways that exhibit complex interactions with vimentin, as demonstrated in numerous studies. Vimentin expression and accompanying EMT were induced upon TGF-β1 stimulation [14,15,16,17], Snail over-expression [18], ZEB2 over-expression [19] and Slug phosphorylation by ERK [20]. 

Interestingly, the *Vim* promoter comprises a binding motif for the key regulators of EMT from the Smad family [15,21], as well as sequences recognized by EMT-related transcription factors NF-κB [22] and AP-1/jun [23]. Moreover, vimentin expression is also transactivated by β-catenin/TCF binding to the *Vim* promoter, thus promoting tumor cell migration/invasion [24].

Epigenetic regulation of vimentin expression has also been shown to play an important role in EMT and cancer progression. It has been demonstrated that vimentin promoter methylation inversely correlates with vimentin expression and disease progression in gastric cancer [25].

Apart from transcriptional regulation of vimentin expression by EMT-related transcriptional drivers, vimentin expression can be regulated also by non-coding microRNAs (miRs). It has been proposed that the HIF-1a–HDAC1 complex transcriptionally inhibits miR-548an expression during hypoxia, resulting in the upregulation of vimentin that facilitates pancreatic tumorigenesis [26]. Likewise, miR-22 [27] and miR-138 [28] were found to oppose EMT by partially suppressing vimentin expression. 

Recent data suggest that Twist, one of the main EMT drivers, promotes EMT not only by E-cadherin suppression but also by negative regulation of vimentin miRs. It has been reported that Twist1 activates the expression of Cullin2 circular RNA (circ-10720), which absorbs miRNA targeting vimentin, leading to increased vimentin mRNA levels [29].

Simultaneously, vimentin itself has a role in modulating EMT signaling. Vimentin levels seem to regulate Snail expression in a feedback loop, and a knock-down of vimentin resulted in decreased Snail1 mRNA levels [12]. Over-expression of vimentin leads to an increase in Slug expression levels, while down-regulation has the opposite effect [10]. Vimentin also regulates Slug by binding to and promoting the activity of ERK, which then phosphorylates Slug [20]. In keratinocytes, the reconstitution of vimentin in vimentin knock-out cells was sufficient to restore ERK1/2 signaling [30]. However, in a different study where cells were plated on laminin-5, the ERK pathway was unaffected after vimentin knock-down [31].

**Figure 1:**
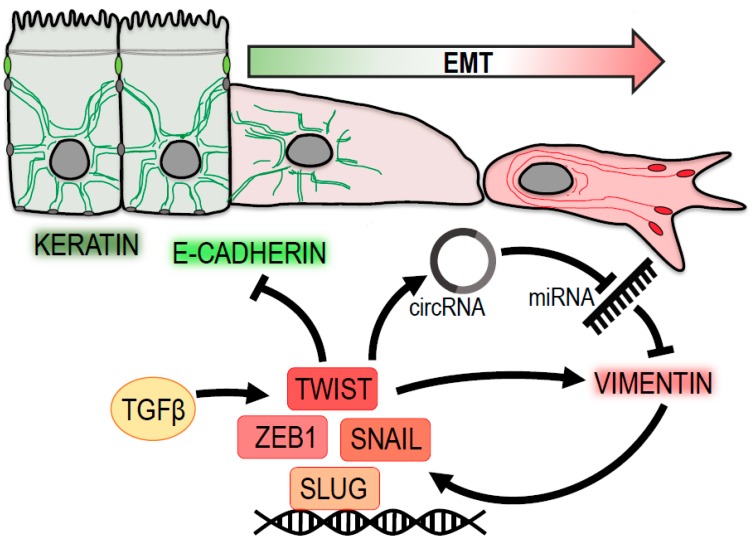
Vimentin at the center of epithelial-to-mesenchymal transition (EMT). Vimentin levels are positively associated with a loss of epithelial traits (green) and a gain of a pro-migratory mesenchymal phenotype (red). Vimentin expression is regulated by transcription factors Twist, Snail, Zeb1 and Slug, which are induced by TGF-β signaling. Twist suppresses the expression of epithelial keratins and E-cadherin. Moreover, it contributes to vimentin upregulation by promoting the expression of circular RNA circ-10720, which suppresses miRNA-mediated downregulation of vimentin. Vimentin itself enhances the expression of pro-mesenchymal transcription factors Snail and Slug.

Taken together, these results place vimentin at the very center of the whole EMT process—both downstream and upstream of major metastatic progression drivers—creating a feedback loop actively supporting the pro-migratory properties of cells (Figure 1).

## 3. Vimentin in the Context of the Cytoskeleton

An essential prerequisite for the fundamental rearrangement of the cytoskeleton in the course of EMT is its coordinated regulation and the interplay of individual cytoskeletal components. Highly organized IF networks are maintained by cytoskeletal linker proteins (cytolinkers) of the plakin protein family (for review see [32,33,34]). Cytolinkers are multimodular proteins that crosslink IFs with microtubules and actin filaments and tether the cytoskeletal network to cell–cell junctions (desmosomes) [35], cell–extracellular matrix (ECM) adhesions (hemidesmosomes and focal adhesions) [36,37], or various intracellular structures (e.g., the surface of the nucleus [38]). While heterogeneous keratin IFs are organized by several plakins (BPAG1 and 2, epiplakin and plectin), the vimentin IF network is mostly controlled by plectin [34,39].

The vimentin IF network has been recently shown to closely associate with other cytoskeletal components to provide a load-bearing “meshwork” supporting the contractile actomyosin system [7]. Vimentin IFs also interact with microtubules through the tumor suppressor APC [40] and plectin [41]. Although the molecular basis for vimentin IF-microtubule linkage is not fully elucidated, it seems that these interactions are instrumental for aligning them or guiding them along each other [10,42]. With actin fibers, vimentin interacts directly by its tail domain [43] and indirectly via crosslinking with plectin [41,44]. For instance, in osteosarcoma U2OS cells, vimentin IFs associate in a plectin-dependent manner with contractile actomyosin arcs and restrict their retrograde flow, thereby regulating the morphogenesis of flat lamellae during migration [45]. Using dermal fibroblasts as a model system, Costigliola et al. [46] showed that vimentin IFs are required for the anisotropic orientation of actomyosin-generated traction stresses propelling single cell migration. 

Importantly, vimentin has been shown to affect the actin cytoskeleton not only mechanistically, by a physical linkage, but also by modulating major actin cytoskeleton signaling pathways. It has been demonstrated that loss the of vimentin IFs integrity, caused either by vimentin deletion, inability of vimentin to polymerase, or its decoupling from other cytoskeletal structures by plectin deletion, leads to increased actomyosin contractility [47,48]. 

Both knock-out and siRNA-mediated depletion of vimentin was shown to increase the activity of RhoA specific Rho GTPase exchange factor GEF-H1 and subsequent RhoA activation and phosphorylation of the myosin light chain, resulting in increased actomyosin contractility [47].

These findings demonstrate that the integrity of vimentin IFs provide a cytoarchitecture with mechanical stability that also enables precise spatiotemporal coordination between all three cytoskeletal components.

## 4. Vimentin in Focal Adhesions

Cooperation of the IFs and actin network is prominent at sites of cell adhesions. In fact, the attachment of cytoskeletal networks to cell adhesions modulates their essential features such as stability [36,49,50,51,52,53], dynamics [36,37,50], or mechanotransduction capacity [48]. Vimentin is found at focal adhesions (FAs), which are dynamic protein complexes interlinking cytoskeleton with ECM that facilitate attachment, generation of traction forces, and migration [11,50,54,55,56,57,58]. The physical linkage between vimentin IFs and FAs strengthens the adhesions [37,56,59] and promotes their dynamics, boosting the migratory potential of cells [37,48].

**Figure 2:**
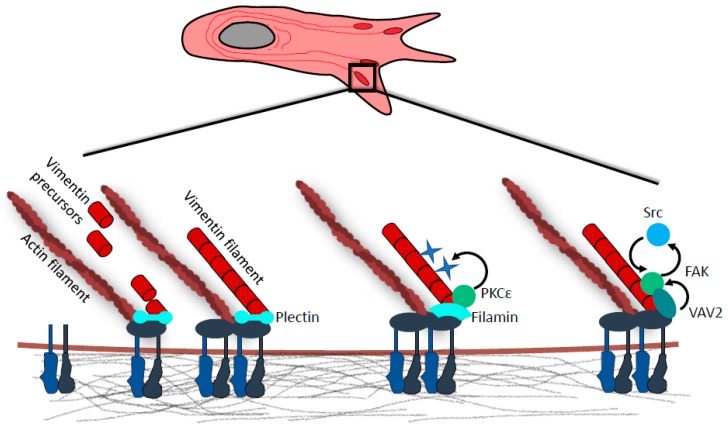
Vimentin in focal adhesions. Vimentin intermediate filaments (IF) precursors are captured at focal adhesions (FAs) via interaction with cytolinker protein plectin. Precursor docking is followed by end-to-end fusion of FA-immobilized and mobile vimentin intermediates with nascent vimentin filaments being incorporated into existing vimentin IF meshwork. Vimentin–FA association is also mediated by cytolinker protein filamin A. Vimentin-bound filamin A serves as a scaffold for PKCε, which phosphorylates vimentin at Serines 6, 38, and 50. Subsequent vimentin IF reorganization favors integrin recycling, formation of cell extensions, and cell spreading. Vimentin IF anchorage to FAs is also required for the recruitment of guanine nucleotide exchange factor (GEF) VAV2. At FAs VAV2 serves as a Rac1 GEF, and active Rac1 then promotes FA assembly through FAK/Src signaling.

Vimentin IF precursors are recruited to FAs via interaction with plectin isoform 1f, where they fuse into nascent filaments [37,60]. These are eventually integrated into a heavily crosslinked IF core, which is in turn stabilized through anchorage to underlying FAs [37] (Figure 2). The recruitment of vimentin to FAs is also dependent on filamin A, which serves as a docking site for PKCε. Interestingly, the phosphorylation of vimentin by PKCε has been shown to be crucial for the delivery of β1-integrin to focal adhesions [61]. The knock-down of either filamin A or vimentin also results in a failure to localize β1-integrin to FAs [57]. In addition, vimentin has been shown to directly bind α2β1 [55] and β3 integrins [62]. Recently, it was demonstrated that the FA protein Hic-5 is important for organization of IFs, as its absence led to a collapse of the IF network [63].

Despite several recent studies addressing the interplay between vimentin IFs and FAs (e.g., [10,46,48,64,65], for review see [66]), the underlying mechanisms remain somewhat enigmatic. Specifically, Terriac et al. revealed, using stimulated emission depletion (STED) microscopy, that vimentin is found in most, but not all, FAs, and that vimentin IFs are localized at large FAs in transformed fibroblasts [58]. Moreover, in fibroblasts, loss of plectin-mediated vimentin IF–FA linkage uncouples the activation of focal adhesion kinase (FAK) from actomyosin-generated tension. This leads to attenuated FAK/Src signaling and reduced activation of downstream MAP kinases Erk1/2 and p38 [48]. Based on this observation, authors proposed a model where FA-anchored IFs impose physical constraints on the actomyosin system to render effective tension transmission and conversion of mechanical stimuli into signaling events [34,48]. In studies using endothelial cells, the size and adhesive strength of FAs were dependent on the expression of vimentin and its presence in the structures [50,56]. However, in a study using oral squamous cell carcinoma cells, authors showed that knock-down of vimentin leads to an increase in β4-integrin levels, and a more adherent behavior when cells were grown on laminin-5. No change in adhesion was observed on fibronectin [31]. These observations, therefore, suggest that the function of vimentin in FAs might be dependent on cell type and the ECM substrate.

Furthermore, vimentin has also been shown to promote FAK activation by recruiting Rac1-GEF VAV2 to focal adhesions [64] (Figure 2). Other studies have shown that both vimentin and plectin form complexes with FAK scaffolding protein RACK1 [67,68]. Loss of vimentin or plectin abrogates RACK1 sequestration on vimentin IF networks, thus dysregulating FAK and PKC signaling [68,69].

Together, these findings contribute to the current understanding that vimentin is a major determinant for FA functions. The relationship between vimentin IFs and FAs is, however, clearly interdependent since mechanical load on FAs modulates vimentin solubility [70] and induces the rearrangement of vimentin IF networks [50].

## 5. Vimentin in Migration and Invasion

Due to its role in EMT, it is not surprising that vimentin plays a pivotal role in the ability of cells to invade their surrounding matrix. This is of particular interest in relation to cancer, where acquisition of a motile phenotype and invasive capacity leads to metastases—the main cause of death in cancer patients.

Multiple studies have investigated vimentin’s role in migration and invasion. Human mammary epithelial cells MCF10A were found to express vimentin in a wound-healing experiment at the wound’s edge in actively migrating cells [71]. Vimentin was also proved to be important in wound-healing assays in works using other cell lines [31,64,72]. Moreover, vimentin knock-down in the lung cancer cell line A549 led to slower and less directed migration. Similarly, oral squamous cell carcinoma cells formed fewer colonies in soft agar and were less capable of invading Matrigel upon vimentin knock-down [31]. Vimentin deficiency in mice resulted in impaired wound healing [73].

The EMT TGF-β pathway and its involvement with vimentin is also implicated in the ability of cells to migrate and invade. The TGF-β1 treatment was sufficient to induce vimentin expression and render the cells capable of wound healing, in the case of epithelial cells [15], and invading through Matrigel, in the case of canine breast cancer cells. This second effect was reversed after prolonged exposure to the stimulus [74]. Other studies focused on the downstream components of the TGF-β pathway. Vimentin was shown to be crucial to the ERK2-Slug-Axl induction of EMT, migration, and 3D matrix invasion. This pathway was also responsible for the ability of cells to extravasate and form metastases in mice [20,75]. The ectopic expression of the downstream target of TGF-β, Snail1, was enhanced vimentin expression and the potential for migration and invasion in prostate cancer cells [76].

The Snail1-Axl-vimentin pathway was found to be downregulated in response to poly ADP-ribose polymerase 1 (PARP-1) inhibition via ILK and GSK3-β. In melanoma cells this led to impaired would healing and loss of capacity to form lung metastases after tail vein injection of melanoma cells into mice. The inhibition of PARP-1 also decreased migration in MDCK cells stimulated to undergo EMT by hepatocyte growth factor (HGF) treatment, but over-expression of vimentin in these cells abrogated that effect, showing that vimentin down-regulation is a key component of the anti-migratory consequence of PARP-1 inhibition [72].

Vimentin also has a function in lamellipodia, branched-actin-rich structures at the leading edge of migrating cells. A recent study showed that phosphorylation by Src and dephosphorylation by SHP2 of Tyr117 of vimentin drives dynamic vimentin IF disassembly and assembly important for lamellipodia dynamics. Tyr117 phosphorylation led to vimentin IF disassembly, the recruitment of the Rac1 GEF Vav2 to the cell membrane, and the induction of lamellipodia formation [77]. Accordingly, in another study, upon Rac1 activation followed by phosphorylation of Ser38 on vimentin, vimentin IFs at the leading edge of mouse embryonic fibroblasts disassembled and retracted, enabling lamellipodia formation [78]. However, a different study showed that lung cancer cells that had vimentin IFs extended into lamellipodia showed more effective migration compared to cells that had vimentin-free lamellipodia [79]. Further studies are therefore necessary to clarify the role of vimentin in lamellipodia formation.

## 6. Vimentin and the Mechanics of 3D Invasion

In particular, vimentin IFs are important for invasion in 3D environments. When invading through the ECM, cancer cells can invade in a collective manner, retaining cell adhesions, or individually as single cells. Single cell invasion can be further classified as proteolytically independent or dependent, known as amoeboid and mesenchymal invasion, respectively [80]. Overall, invasion in confined environments requires orchestration of cytoskeletal tension, dynamics and reorganization not only for persistent locomotion but also to cope with resisting elastic and frictional forces against the cell surface [81]. By virtue of the unique mechanical properties of vimentin [82], vimentin IFs provide transformed cells with elasticity [83] and contribute to the vicious cycle of tumor tissue stiffening [84,85]. Consistently, Liu et al. [10] showed recently that highly invasive breast carcinoma cells devoid of vimentin are more pliant, less contractile and lose directional persistence of migration. Moreover, vimentin was shown to be indispensable for generation of compartmentalized pressure that drives 3D cell migration [86]. 

From a mechanistic point of view, vimentin IFs were shown to confer resistance to deformation in migrating cells. Atomic force microscopy and microfluidic optical stretcher experiments revealed that vimentin knock-down MDA-MB-231 cells were significantly more deformable and stretchable [12]. This is likely due to vimentin IFs assuming a front–rear polarity, forming a central cage-like structure surrounding the nucleus with individual filaments extending to the trailing edge of the cell [37,78]. 

**Figure 3:**
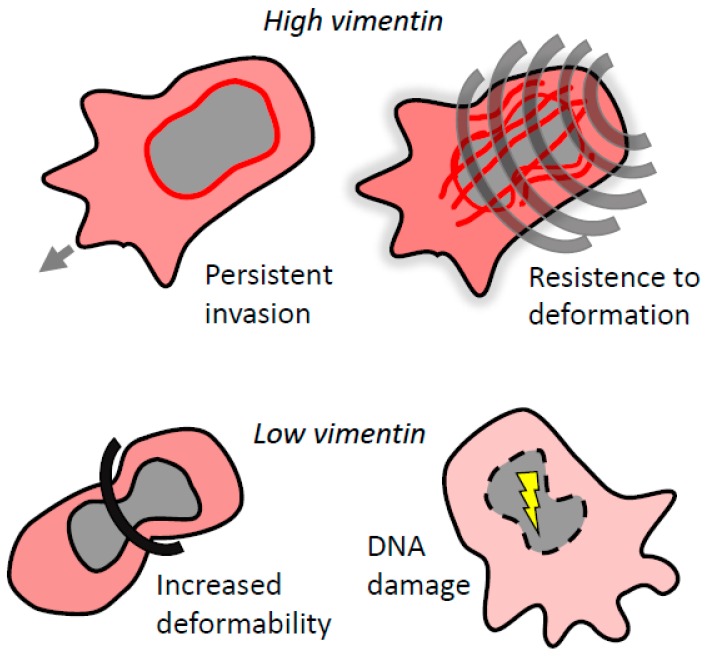
Vimentin regulates invasion in confined environments. Vimentin protects the nucleus from mechanical stress by controlling nuclear deformability. High levels of vimentin provide cells with enhanced resistance to deformation and lead to persistent migration. Low levels of vimentin result in loss of nuclear integrity and DNA damage, which abrogates invasion. However, in some cases, loss of vimentin can promote bleb-based invasion.

Protease-independent amoeboid cells, in particular, have to cope with various mechanical obstacles and dynamically deform their cell body shape, including the cell nucleus. It was recently shown that vimentin regulates nuclear shape and volume and protects the nucleus from DNA damage during invasion in confined environments. In amoeboid dendritic cells, vimentin IFs along with actin filaments were necessary for protection of the nucleus during confined migration, and loss of vimentin resulted in defective migration [87]. However, in mouse embryonic fibroblasts, the absence of vimentin IFs promoted migration through small pores [88]. Moreover, loss of vimentin enhanced amoeboid leader bleb-based migration of confined cancer cells [89]. Together these studies suggest that the contribution of vimentin IFs to perinuclear stiffness is required for the preservation of nuclear integrity and constitutes a critical parameter for controlling confined migration (Figure 3). Interestingly, vimentin IFs were also shown to deform the cell nucleus by forming rings around it during cell adhesion [90]. 

Vimentin further regulates important structures for mesenchymal invasion—actin-rich protrusions, termed invadopodia, that are sites of matrix degradation [91]. Vimentin IFs were shown, using transmission electron microscopy, to extend into the bodies of mature invadopodia in cells invading a 3D matrix. Vimentin expression was required for invadopodia elongation and maturation [92]. Later on, Yoneyama et al. observed that vimentin filaments bind to invadopodial actin via plectin at the bases of invadopodia. This link, along with vimentin filament formation, was necessary for invadopodia formation in Matrigel, secretion of matrix metalloproteinases (MMPs), and, therefore, the capacity for matrix degradation, invasion, and lung metastasis formation in mice [93].

The delivery of MT1-MMP, a key MMP, to the membrane, and resulting invasion through 3D collagen gel, was also shown to be dependent on vimentin cleavage by calpain in a response induced by the S1P sphingolipid in endothelial cells [94]. Interestingly, a converse effect of sphingolipids was shown in a variety of cell lines (MEFs, MDA-MB-231 and C643), where S1P and sphingosylphosphorylcholine (SPC) triggered ROCK-dependent serine phosphorylation and vimentin filament disassembly and collapse to the region around the nucleus leading to the loss of migration [95].

A study by Messica et al. looked into the differences in the effect of regulating vimentin expression on singe-cell versus collective migration. They observed no change in the trajectories of single cells in the case of vimentin silencing in highly invasive MDA-MB-231 cells, yet these cells were less effective at migrating collectively. Severe defects were observed upon vimentin knock-down in both a transwell migration assay and an invasion assay. Interestingly, the cells were more effective at migrating when they were more densely plated on the membrane, although they migrated through it individually [12]. The role of vimentin in collective migration was also investigated in astrocytes where it appeared that vimentin IFs regulated the distribution of traction forces and the maintenance of cell–cell interactions, thus contributing to successful collective migration [65]. In collective migration induced by interstitial fluid pressure, vimentin was among the upregulated genes [96]. However, how vimentin might aid in migration in the context of the density of cells is still unclear. 

## 7. Research Focused on Vimentin in Metastases

Having discussed the diverse regulation of vimentin and its involvement in migration and invasion, the following two studies underline the crucial role vimentin plays in metastasis formation. In a study by Liu et al., metastatic and parental non-metastatic cell lines of the same origin—oral squamous cell carcinoma—were compared and vimentin was identified as the protein with the most increased expression in the metastatic cell line relative to the parental one. They also showed, by immunohistochemical staining of oral squamous cell carcinoma samples, that a high amount of lymph node metastases correlated with high vimentin expression [17].

Simultaneously, it seems that vimentin does not need to be present in the invading cancer cells to facilitate metastasis formation. As Richardson et al. demonstrate, it can occur through the tumor microenvironment. Investigation of a full-body vimentin knock-out (VIM^−/−^) in a described mouse model of lung adenocarcinoma carrying the *LSL-Kras^G12D^* and *Lkb1^fl/fl^* driver mutations revealed that loss of vimentin had no effect on the development of primary tumors. However, lymph node metastases were reduced by half. Upon closer analysis, in VIM^+/+^ mice, vimentin was absent from tumor cells but present in the cancer associated fibroblasts (CAFs) that surrounded the groups of cells that had broken off from the primary tumor, termed collective invasion packs. In the vimentin knock-out mice, the number of these cell clusters decreased but the number of cells per pack remained the same. The presence of CAFs, which was not observed in VIM^−/−^ mice, was the main difference in the microenvironment of tumors between the two mouse models. In 3D in vitro invasion assays, the addition of CAFs to lung adenocarcinoma cell line spheroids increased spheroid branching. The knock-down of vimentin in CAFs decreased this effect, meaning that the presence of CAFs with intact vimentin led to effective collective migration [97]. This highlights the importance of vimentin in other cell types associated with tumors and metastases and the variety of roles vimentin plays in metastasis formation.

## 8. Vimentin as a Drug Target

The majority of deaths in cancer patients are directly or indirectly caused by metastases [98]. It is, therefore, essential to develop effective anti-metastatic treatment. Cancer cell invasiveness, directed cancer cell motility through the extracellular matrix, is the first and essential step of the metastatic process [80]. For that reason, migrastatics, a novel category of cancer drugs targeting cancer cell motility, represent excellent candidates for anti-metastatic drugs [99].

The importance of vimentin in EMT and other processes involved in cell motility makes it an attractive migrastatic drug target. However, unlike in the case of the actin or the microtubule cytoskeleton, there is currently no routinely used drug to specifically target intermediate filaments. This makes their study, as well as their use as a target in clinic, much more challenging. In the past years there have been several studies, discussed in the following text, promising the possibility to specifically target vimentin and, thus, potentially affect cancer progression (Table 1). 

Probably the most thoroughly investigated drug targeting vimentin IFs is the tumor inhibitor Withaferin A (WFA), derived from the plant *Withania somnifera* [120], which was found to target and directly bind vimentin in an effort to find the mode of action related to its antiangiogenic properties [123]. However, WFA also has many other molecular targets, including STAT1 and STAT3, Notch1, FOXO3A and kinases PKC, p38, JNK, Akt and ERK, as reviewed by Vanden Berghe et al., 2012 [124]. WFA inhibits tumor growth and has pro-apoptotic activity [120,125].

WFA downregulates vimentin expression and induces the disassembly of vimentin filaments in a dose dependent manner. In doses of 500nM and lower, which are not pro-apoptotic nor cytotoxic, WFA seemed not to alter vimentin expression levels and led to vimentin Ser56 phosphorylation. Here, vimentin collapsed to the perinuclear space and maintained strong anti-migratory and anti-invasive properties in cancer cells. No effects were observed in normal human keratinocytes [121]. In higher doses, WFA changed the gene expression profiles of treated cells, which indicated the reversal of EMT. The changes in vimentin expression levels were lower but still detectable when WFA was administered in the xenograft mouse model [119]. Apoptosis induction by WFA seemed to be completely vimentin independent and migration inhibition partly so, as WFA further inhibited motility even in vimentin knock-down cells [126].

There are several drugs that induce apoptosis in a vimentin-dependent manner. Arylquin 1, a derivative of 3-arylquinoline, associates with vimentin, displacing the prostate apoptosis response-4 (Par-4). This leads to its secretion and paracrine apoptosis induction in tumor cells [101]. Simvastatin selectively induces apoptosis in vimentin-containing cells and causes vimentin filament disassembly and bundling around the nucleus [114]. Another statin, Fluvastatin, was cytotoxic to invasive MDA-MB-231 cells, but not epithelial MCF-10A cells. Fluvastatin treatment resulted in vimentin degradation in a caspase-3-dependent manner [104]. The Plk1 inhibitor volasertib is a drug that induces apoptosis and also inhibits migration. Plk1 inhibition leads to a decrease in vimentin Ser82 phosphorylation and the corresponding loss of cMet phosphorylation via β1-integrin [118].

As expected, drugs inhibiting or reversing EMT also have an effect on the vimentin IF network. Silibin, resveratrol, and dioscin all suppress vimentin expression and decrease migration and invasion [102,108,112,113]. Ursolic acid has these effects and also reduces tumor cell growth and induces apoptosis [116,127]. In a similar fashion, a component of ginseng, Ginsenoside 20(R)-Rg3, has been found to prevent EMT upon TGF-β stimulation, repressing vimentin among other EMT markers [105]. The garlic component ajoene binds vimentin directly and condenses the filament network, inhibiting migration and invasion in a vimentin-dependent manner [100]. Salinomycin, a potassium ionophore, was found to selectively target E-cadherin knock-down breast cancer cells. Those cells underwent EMT and expressed cancer stem cell (CSC) markers [128]. Salinomycin drastically reduced the CSC population in colorectal cancer cells, which also showed downregulated vimentin levels, EMT reversal, and decreased migration [110]. In ovarian cancer cells, the effect of salinomycin on vimentin and EMT was achieved through repression of the Wnt/β-catenin pathway [111], which also induces EMT and vimentin expression [24].

A promising option for targeted cancer therapy is aptamers, single-stranded RNA or DNA molecules that bind to specific peptides. Their significant advantage is their high specificity for targets [129]. A vimentin targeting DNA aptamer NAS-24 caused apoptosis of cancer cells and reduced adenocarcinoma tumors in mice [106]. The RNA aptamer P15 was found to bind vimentin on the cell surface and specifically target pancreatic adenocarcinoma cells. It was internalized by the cells and inhibited migration while having no effect on cell proliferation [107]. P15 is therefore a potential migrastatic molecule.

In a similar manner to P15, the monoclonal antibody 86C bound to cell surface vimentin and specifically targeted glioblastoma cells. 86C binding led to the endocytosis of vimentin, caspase-3 activation, and apoptosis induction in multiple glioblastoma cell lines. 86C was tested in vivo, where the treatment decreased tumor growth in mice [122].

Probably the closest to a vimentin-specific anti-cancer drug is the recently discovered FOXC2-inhibiting vimentin effector 1 (FiVe1) compound. This was identified in a high throughput screen for compounds that selectively and irreversibly inhibit the growth of mesenchymally transformed breast cancer cells and soft tissue sarcomas of various histological subtypes [103]. FiVe1 directly interacts with vimentin, promotes the collapse of vimentin architecture and vimentin degradation leading to the morphological rearrangement into a more epithelium-like state in mesenchymally transformed cells. FiVe1 treatment leads to vimentin phosphorylation and disorganization during metaphase, ultimately resulting in mitotic catastrophe. In contrast to other mitosis-inhibiting compounds that target mitosis in all cells (e.g., taxol), FiVe1 compound treatment was shown to have the advantage of selectively targeting only vimentin-expressing cells, thus reducing toxicity to rapidly dividing epithelial tissues and enabling the possibility to reduce the unwanted side effects of anticancer therapies. However, medicinal chemistry modifications of the FiVe1 compound or a discovery of similar vimentin-targeting drug will still be needed in order to improve the potency and pharmacological properties of the compound to increase the translational potential of vimentin targeting as a therapy for mesenchymal cancers. 

## 9. Concluding Remarks

In recent years, there is growing evidence that vimentin IFs unequivocally belong to the major EMT initiators and, as such, play a key role in tumor progression and dissemination (Figure 1). The results reviewed in this text indicate that inhibition of vimentin has the potential to decrease cell migration, proliferation, or invasion. By modulating the dynamics and mechanotransduction potential of focal adhesions (Figure 2), vimentin deletion in cells results in slower and less directional migration in 2D and 3D environments. Therefore, new approaches targeting vimentin constitute promising therapeutic venues for anti-cancer therapy. 

On the other hand, manipulating vimentin IFs often induces cytoskeletal compensations leading to increased actomyosin contractility. Higher cell contractility, paired with higher cell elasticity, can be beneficial for the ability of various cell types to migrate, in particular in the context of invasion through environments with higher degrees of confinement (Figure 3). Compensatory effects, such as those linked to the actin cytoskeleton, should therefore be taken into consideration while designing vimentin-based anti-cancer strategies. 

Vimentin is a multifunctional protein, with diverse binding partners and functions, many of which remain to be elucidated. For example, apart from the earlier mentioned roles of vimentin in IFs and FAs, and as transcriptional regulator of EMT processes, vimentin is also secreted as an extracellular protein and shown to have various functions, including regulation of axonal growth [130] or spheroid formation of glioblastoma cancer stem cells [122]. In addition, growing evidence suggests its role as a cancer biomarker [131,132]. 

Due to the numerous roles of vimentin, the effect of vimentin inhibition in non-malignant cells should be considered. To this end, consequences of its inhibition should be studied in animal models and effects on processes entailing cell migration (such as wound healing [73,133] or immune response [87,134]) should be carefully examined. However, since mice lacking vimentin develop and reproduce without any major problem [135], we can anticipate that beneficial vimentin inhibition in malignant cells outweighs any potential adverse effects. Although lack of high-affinity and high-specificity ligands hindered the development of specific vimentin-targeting therapeutics over the past years, several studies turned up promising candidates. To date, only one—FiVe1—shows the real potential of specific vimentin targeting suitable for future cancer therapy. Despite these first promising results, further research and development efforts are required to understand the complexity of vimentin IF targeting in order to develop successful targeted therapy.

## Figures and Tables

**Table 1:** Vimentin targeting compounds.

Compound	Mode of Action	Effect	Direct Binding	Origin	Cancer-Related Clinical Trials *	References
**Aojene**	Vimentin IF collapse	Anti-invasive/migratory	Yes	*Allium sativum* (garlic)	-	[100]
**Arylquin 1**	Par-4 displacement from vimentin, Par-4 secretion	Paracrine apoptosis induction in cancer cells	Yes	3-arylquinoline derivative		[101]
**Dioscin**	Suppression of vimentin expression via TGF-β1 pathway	EMT reversal, anti-invasive/migratory	NK	steroid saponin isolated from Chinese medicinal plants	-	[102]
**FiVe1**	Ser56 phosphorylation, vimentin IF collapse	MET, mitotic catastrophe in vimentin-containing cells	Yes	Unknown	-	[103]
**Fluvastatin**	Caspase-3-mediated proteolysis of vimentin	Cytotoxicity in invasive cancer cells	NK	synthetic indole-heptanoic acid derivative	Phase 1-2	[104]
**Ginsenoside 20(R)-Rg3**	Suppression of vimentin expression via TGF-β1 pathway	EMT suppression	NK	*Panax ginseng* (ginseng)	Phase 2	[105]
**NAS-24**	Unknown	Apoptosis induction in cancer cells	Yes	synthetic	-	[106]
**P15**	Unknown	Anti-invasive/migratory	Yes	synthetic	-	[107]
**Resveratrol**	Suppression of vimentin expression via TGF-β1 pathway	Anti-invasive/migratory, metastasis reduction	NK	phytoalexin found in red grapes and other plants	Phase 1-2	[108]
**Salinomycin**	Suppression of vimentin expression via Wnt/β-catenin	MET, anti-migratory, CSC reduction	NK	*Streptomyces albus*	-	[109,110,111]
**Silibinin**	Suppression of vimentin expression	EMT suppression, anti-invasive/migratory, tumor dissemination and growth reduction	NK	*Silybum marianum*	Phase 1-2	[112,113]
**Simvastatin**	Vimentin IF collapse	Apoptosis induction in vimentin-containing cells	NK	derivative of afermentation product of *Aspergillus terreus*	Phase 1-3 (FDA approved for Type II diabetes)	[114,115]
**Ursolic acid**	Suppression of vimentin expression possibly via TGF-β1 pathway	Anti-invasive/migratory, tumor growth reduction, apoptosis	NK	pentacyclic triterpenic acid found in plants	-	[116]
**Volasertib**	Decrease in Ser82 phosphorylation caused by Plk1 inhibition, loss of cMet phosphorylation via β1-integrin	MET, anti-invasive/migratory, apoptosis	NK	dihydropteridinone derivative	Phase 1-3	[117,118]
**Withaferin A**	Doses below 500 nM: Ser56 phosphorylation, vimentin IF collapse Higher doses: vimentin expression lowered	Dose below 500 nM: anti-invasive/migratory in cancer cells Higher doses: MET, apoptosis induction (vimentin independent)	Yes	*Withania somnifera*	-	[119,120,121]
**86C**	Internalization of cell surface vimentin, otherwise unknown	Apoptosis induction, tumor growth reduction	Yes	monoclonal antibody	Phase 1	[122]

* source data: https://clinicaltrials.gov/; NK = not known; IF = intermediate filaments, EMT = epithelial-to-mesenchymal transition; MET = mesenchymal-epithelial transition.

## List of Abbreviations:

Akt	Akt kinase a.k.a. Protein kinase B
AP-1	Activator protein 1
APC	Adenomatous polyposis coli
CAFs	Cancer-associated fibroblasts
CSC	Cancer stem cell
ECM	Extracellular matrix
EMT	Epithelial–mesenchymal transition
ERK	Extracellular signal–regulated kinase
FA	Focal adhesion
FAK	Focal adhesion kinase
FOXO3A	Forkhead box O3
GEF	Guanine nucleotide exchange factor
GSK	Glycogen synthase kinase
HDAC	Histone deacetylase
HIF-1	Hypoxia-inducible factor
IFs	Intermediate filaments
ILK	Integrin-linked kinase
JNK	c-Jun N-terminal kinase
MAPK	Mitogen-activated protein kinase
MEFs	Mouse embryonic fibroblast
MET	Mesenchymal–epithelial transition
NF-κB	Nuclear factor kappa-light-chain-enhancer of activated B cells
p38	p38 MAPK
PKC	Protein kinase C
Plk	Polo-like kinase
RACK1	Receptor for activated C kinase 1
ROCK	Rho-associated protein kinase
S1P	Sphingosine-1-phosphate
SHP2	Protein-tyrosine phosphatase shp2
Snail	Zinc finger transcription repressor Snail
SPC	Sphingosylphosphorylcholine
Src	Protein-tyrosine kinase Src
STAT	Signal transducer and activator of transcription
STED	Stimulated emission depletion
TCF	Transcription factor TCF
TGF-β	Transforming growth factor beta
Twist	Zinc finger transcription repressor Twist
VAV2	Guanine nucleotide exchange factor VAV2
WFA	Withaferin A
ZEB	Zinc finger E-box-binding homeobox
